# Critically Appraised Topic on Adverse Food Reactions of Companion Animals (8): Storage Mites in Commercial Pet foods

**DOI:** 10.1186/s12917-019-2102-7

**Published:** 2019-10-31

**Authors:** Thierry Olivry, Ralf S. Mueller

**Affiliations:** 1 Department of Clinical Sciences, College of Veterinary Medicine, North Carolina State University, 1060 William Moore Drive, Raleigh, NC 27607 USA; 2 Medizinische Kleintierklinik, Centre for Clinical Veterinary Medicine, Ludwig Maximilian University, Veterinärstrasse 13, 80539 Munich, Germany

**Keywords:** Atopic dermatitis, *Dermatophagoides*, Dog, Food allergy, Forage mite, House dust mite, Pet food, Storage mite, *Tyrophagus*

## Abstract

**Background:**

Dogs with year-round atopic dermatitis are often sensitized to *Dermatophagoides* house dust mites (HDM). Storage mites (SM) are known to grow on cereal-rich foods. *Tyrophagus* SM can exacerbate clinical signs of allergy in laboratory dogs sensitized to HDM. Consequently, atopic dogs with high-levels of HDM-specific IgE are likely to have a flare of signs after eating a food contaminated with SM; the development of such flares would lead to a false positive diagnosis of food allergy. Herein, we reviewed the published evidence about the growth of SM on commercial dry pet foods.

**Results:**

We searched two databases on January 25, 2019 for articles providing original information on the growth of SM on commercial dog foods. We found ten articles, five reporting results of laboratory experiments and five from field studies. Storage mites, especially *Tyrophagus putrescentiae*, can multiply on protein- and fat-rich dog foods. The population growth is higher when the initial mite density is high and when kibbles are crushed. When storage conditions lead to the overgrowth of molds on the kibbles, the mite proliferation is higher. Storage mites do not bore holes in food packages but invade bags via defective seals. In the field, SM contamination usually is undetectable in newly-opened commercial dog foods, and, if present, their number is low. When newly-purchased bags are stored in temperate conditions indoors, little overgrowth—if any—of SM occurs. However, when kept in environmental conditions with higher temperature and humidity, *Tyrophagus* mites will enter and proliferate in sealed food packages.

**Conclusions:**

Commercial dry pet foods should be kept indoors and sealed to decrease the risk of contamination with SM. When performing dietary restriction (elimination) and provocation trials for the diagnosis of food allergies in dogs, it seems preferable to choose newly-purchased bags—of both original and testing diets—to reduce the probability of their contamination with SM, especially *Tyrophagus putrescentiae.* In case of doubt about the presence of SM in any of these foods, one should perform food challenges with single home-cooked ingredients. Storage mite contamination might lead to an erroneous diagnosis of food allergy in HDM-sensitized dogs.

## Background

Atopic dermatitis (AD) is a common, chronic, recurrent pruritic allergic skin disease of dogs; it has, in most patients, a characteristic skin lesion distribution [1]. Canine AD is most often associated with the production of IgE specific for environmental and/or food allergens [2, 3]. Worldwide, house dust mites (HDM) of the *Dermatophagoides* genus are the most common allergens recognized by the circulating IgE of atopic dogs (reviewed in [4]). Storage mites (SM) represent another group of acarids that commonly invade food sources, especially cereals. Frequently-encountered SM species are *Acarus siro*, *Lepidoglyphus destructor*, *Glycyphagus domesticus* and *Tyrophagus putrescentiae* [4]. An IgE reactivity against SM is also very common in dogs with AD [5, 6]. The extensive cross-reactivity that exists between HDM and SM allergens implies that HDM-specific IgE from sensitized atopic dogs are likely to also recognize homologous allergens in SM, and vice-versa [7, 8]. Such allergen cross-reactivity is probably clinically-relevant, as beagles experimentally-sensitized to the *Dermatophagoides farinae* HDM exhibited a flare of clinical signs when environmentally- or orally-challenged with the SM *Tyrophagus putrescentiae* [9]. Consequently, atopic dogs with high-levels of *Dermatophagoides farinae* HDM-specific IgE are likely to have a flare of clinical signs if eating a food item contaminated with SM; such recurrence of signs would lead to a false positive diagnosis of food allergy.

## Clinical scenario

A three-year-old male West Highland white terrier living in Florida has a two-year history of continuously-deteriorating skin lesions and pruritus affecting the axillae, groin, ventral neck and paws. You recently made the diagnosis of nonseasonal AD. Both allergen-specific IgE serology and intradermal testing confirmed the reactivity to the *Dermatophagoides farinae* HDM. An 8-week elimination diet performed with an extensively hydrolyzed dog food led to a noticeable—yet partial—improvement of signs; these worsened after provocation with the previously fed diet. Further challenges with individual components of that first diet did not cause flares, however. You wonder if this discrepancy in challenge results could be due to HDM-cross-reactive SM present in the original diet and that, after all, your patient might not have a food allergy.

## Structured question

Are SM present in commercial pet foods?

## Search strategy

We searched the Web of Science Core Collection and CAB Abstract databases on January 25, 2019 with the following string: (dog or dogs or canine or cat or cats or feline) and ((storage and mite*) or *Acarus* or *Tyrophagus* or *Lepidoglyphus* or *Glycyphagus*)) and (food* or diet*). There were no restrictions for publication dates or languages. We did not consider conference abstracts or review papers because of our need for detailed study results. Finally, we scanned the bibliography of each selected article for additional references relevant to our question.

## Identified evidence

Our search of the Web of Science and CAB abstracts identified 54 and 33 articles, respectively*.* Among these, we selected ten papers [10–19], eight of these common to both database searches. The scanning of the bibiography of each paper did not provide any additional publication relevant to our topic. Articles reported results from either laboratory [10, 14, 17–19] or field studies [11–13, 15, 16], the latter conducted in the USA [11], Germany [12], Spain [13], Scotland [15] and Australia [16].

## Evaluation of evidence

### Laboratory studies

There were five articles reporting results from laboratory studies, and these are summarized chronologically; all results are summarized in the online Additional file 1: Table S1.

In 1972, Sinha and Paul were the first to report on the survival and multiplication of mites in dry dog foods [10]. *Dermatophagoides farinae* HDM and *Glycyphagus domesticus* SM were inoculated onto four commercial dry dog foods and other substrates; the authors observed the growth of these mites for a little over 2 months. While the *Dermatophagoides* HDM flourished and multiplied on all four dog foods, these did not support the multiplication of the *Glycyphagus* SM.

Nearly 40 years later, Canfield and Wren tested the ability of the SM *Tyrophagus putrescentiae* to survive and grow on three commercial dry dog foods [14]. Kibbles were inoculated with ten female mites and observed for 5 weeks with molds allowed to grow onto half of the samples. *Tyrophagus* mites grew on all three dog foods, with the highest numbers of mites found whenever molds had been allowed to grow on the kibbles.

In 2015, Hubert and colleagues evaluated the capability of *Tyrophagus putrescentiae* to infest and proliferate on samples of dog foods stored in nine different sealed plastic bags and a lidded cup [17]. Mites were placed in the vicinity of the closed food packages for 3 months. After that time, *Tyrophagus* SM were discovered in 5/9 bags (55%), with the mites most often discovered in bags made of polypropylene or polyethylene film monolayers; mites had not made holes in the packaging itself but had entered the bags via faulty seals. Lidded cups were not contaminated.

The same year, investigators from the same laboratory in the Czech republic evaluated whether *Tyrophagus putrescentiae* SM preferred to grow on protein-, fat- or carbohydrate-rich diets [18]. Mites were first adapted on either a protein- and fat-rich commercial dog food or a low-protein, low-fat but carbohydrate-rich wholemeal spelt flour (see Supplementary material 1 for diet details). After 6 months, diets were changed twice, 4 weeks apart. *Tyrophagus* storage mites adapted for 6 months on either diet grew best on the dog food richer in proteins and fat rather than the flour richer in carbohydrates.

Finally, in 2016, the same investigators tested the growth of *Tyrophagus putrescentiae* for 4 weeks on samples from a single commercial dog food in different conditions [19]. In the first experiment, mites were found to grow better on the green and brown rather than the white and red dog food kibbles, but whether these kibbles of different colors had the same nutrient composition was not specified. In the second study, the mite growth rate was higher if kibbles were crushed rather than intact, and when the initial mite population density was highest (100 mites). In the third, four different strains of *Tyrophagus* mites grew better on the crushed dog food compared to an HDM-rearing diet. The final experiment confirmed that, whatever the strain of *Tyrophagus*, the higher the initial mite inoculum (i.e., 100), the higher the final mite count.

### Field studies

We found five articles that reported the results of field studies investigating whether or not HDM or SM were present in commercial dog foods: two studies were purely descriptive [11, 12] while the other three investigated the presence of mites in different experimental conditions [13, 15, 16]. Again, we will describe the study results in chronological order.

In the first study, DeBoer and Schreiner tested whether or not the HDM *Dermatophagoides farinae* contaminated dog foods purchased in midwestern region of the United States [11]. The test material consisted of 30 purchased and 50 pet owner-obtained commercial dry dog food samples. The HDM contamination was determined using an ELISA for the group II *Dermatophagoides* allergens, and none was detected.

Similarly to the results above, Henneveld et al. tested 23 different bags of commercial dry dog foods for mite contamination over a consecutive 6-week period in Germany [12]. Even though the bags were open twice daily (and closed thereafter) to feed the dogs, SM were not discovered, by microscopic examination of flotation samples [20], in any of the samples examined.

In 2008, Brazis and colleagues were the first to report on the influence of different storage conditions on the contamination of dry dog foods with SM in Spain [13]. Ten commercial dry dog foods were left open while three of them were also sealed; duplicate bags were either kept in a laboratory or stored in a ventilated garage with outdoor access for 6 weeks. At the beginning of the study, the investigators found a low number of mites (one mite fragment and two *Acarus siro*) in 2/10 bags of dog foods (20%). Under laboratory storage conditions in a low average temperature (16 °C) and humidity (68%), mites were not detectable for up to 6 weeks using two different methods. In contrast, when bags were stored in a garage with high temperatures (average: 23 °C) and humidity (average: 71%) for 6 weeks, *Tyrophagus* mites were found in 8/10 open bags (80%) and in 2/3 (67%) of the sealed replicates by the flotation technique, the most sensitive detection method.

In 2011, Gill and others stored identical bags of a single commercial dry dog food in ten different households in Scotland [15]. Bags were divided equally between the original sack with its reusable seal, a paper bag whose top was rolled for closure and a plastic box with a sealed lid. These replicates were stored next to each other, and the food was sampled every month for SM detection. After 3 months, mite numbers were significantly higher in the food samples stored in paper bags compared to baseline: 6/10 paper bags had detectable mites, either *Dermatophagoides* or *Tyrophagus*; four and one mites were found in three (30%) sealable plastic bags and one of ten (10%) plastic boxes, respectively. There was no significant association between the temperature or relative humidity and the mite numbers.

In the last field study, dog owners in eastern Australia provided 20 samples of commercial dry dog foods stored in open bags or storage boxes in home environments [16]. The food samples were examined for the presence of SM, and a small portion was kept for two additional months before their incubation under higher humidity and temperature conditions. Finally, nine new bags of commercial dog foods were purchased and tested like for field samples above. Altogether, mites were undetectable in any specimens after any of the incubation times. Similarly, SM were not observed when opening newly-purchased bags and after storing the foods for 6 weeks at room temperature. In contrast, when incubating samples of these foods at high temperature (26 °C) and humidity levels (80%), SM were present in 2/9 samples (22%) as early as 3 weeks after beginning the experiment; after 6 weeks of incubation, 7/9 foods (78%) had detectable mites identified as *Tyrophagus putrescentiae.*

## Conclusion and implication for practitioners

Even though our search strategy only identified a small number of studies, their results were usually concordant, and there are several logical conclusions to make.

Storage mites, especially for the most commonly found and studied *Tyrophagus putrescentiae*, can grow in protein- and fat-rich dog food kibbles [14, 18, 19]. Their population growth is higher when the initial contaminating mite density is high [19], when kibbles are crushed [19] or when suboptimal storage leads to mold overgrowth [14]. When purchasing commercial dry dog foods, SM contamination is not detectable [12, 16], but, when present, the mite number is very low [13]. When storing bags in temperate conditions indoors, one can expect little overgrowth—if any—of SM. In contrast, when keeping dry dog foods in situations with high temperatures and humidity, for example in a garage, *Tyrophagus* mites will enter and proliferate in the packages [13, 16]. In such conditions, SM can also contaminate sealable bags [13], which they will invade via faulty seals [17].

In conclusion, it is best to keep commercial dry dog foods indoors and in temperate conditions and to seal bags to decrease the risk of their contamination with *Tyrophagus* SM. When performing restriction (elimination) and provocation trials for the diagnosis of food allergies in dogs (and likely cats), it seems preferable to choose newly-purchased bags—of both testing and original diets—to reduce the probability of their contamination with *Tyrophagus putrescentiae.* Indeed, the allergenic cross-reactivity between the *Tyrophagus* SM and the *Dermatophagoides* HDM is very high [6, 8], and such cross-reactivity could affect the clinical interpretation of dietary trials in atopic dogs sensitized to HDM [9]. Indeed, atopic dogs with high-levels of *Dermatophagoides farinae* HDM-specific IgE are likely to have signs flaring after eating a food contaminated with SM, thus leading to a false positive diagnosis of food allergy. In case of doubt about pet food contamination with SM, one should perform dietary challenges with home-cooked ingredients previously preserved indoors and in temperate conditions.

As it appears that the main mite naturally growing on commercial dry dog foods is *Tyrophagus* and this SM is the only one whose cross-reactivity with HDM was shown to be clinically-relevant, one should reconsider the validity and clinical relevance of performing IgE serological and intradermal testing with the other less common SM species.

## Future research needs

*Tyrophagus* mites can trigger flares of allergy in laboratory beagles experimentally-sensitized with the *Dermatophagoides* HDM [9]. Whether or not such recurrence of signs also would occur in HDM-reactive dogs with spontaneously-arising AD needs to be confirmed. If such AD flare were to occur, it would be worthwhile to determine the range of SM density that would result in a worsening of signs in dogs with variable levels of HDM- and SM-specific IgE.

Whenever the humidity and temperature are high and the storage of pet foods cannot be controlled effectively, the use of mite traps [21] or the supplementation of pet foods with insect growth regulators/acaricides would be worth of further studies to limit the contamination of these diets with SM. Indeed, S-methoprene and spinosad have been shown to inhibit—albeit slowly—the growth of *Tyrophagus putrescentiae*, and dogs can ingest these two chemicals with no apparent toxicity [22].

## Supplementary information

**Additional file 1: Table S1.** Summary of articles reporting information on storage mites in commercial dog foods.

## Data Availability

All results summarized in this article are available in Additional file 1: Table S1.

## Abbreviations

AD: Atopic dermatitis
HDM: House dust mites
SM: Storage mites
